# Little information loss with red-green color deficient vision in natural environments

**DOI:** 10.1016/j.isci.2023.107421

**Published:** 2023-07-18

**Authors:** David H. Foster, Sérgio M.C. Nascimento

**Affiliations:** 1 Department of Electrical and Electronic Engineering, University of Manchester, Manchester M13 9PL, UK; 2 Physics Center of Minho and Porto Universities (CF-UM-UP), University of Minho, 4710-057 Braga, Portugal

**Keywords:** Natural sciences, Physics, Biological sciences

## Abstract

Inherited color vision deficiency affects red-green discrimination in about one in twelve men from European populations. Its effects have been studied mainly in primitive foraging but also in detecting blushing and breaking camouflage. Yet there is no obvious relationship between these specific tasks and vision in the real world. The aim here was to quantify the impact of color vision deficiency by estimating computationally the information available to observers about colored surfaces in natural scenes. With representative independent sets of 50 and 100 hyperspectral images, estimated information was found to be only a little less in red-green color vision deficiency than in normal trichromacy. Colorimetric analyses revealed the importance of large lightness variations within scenes, small redness-greenness variations, and uneven frequencies of different colored surfaces. While red-green color vision deficiency poses challenges in some tasks, it has much less effect on gaining information from natural environments.

## Highlights

• Discrimination tasks do not predict real-world impact of color vision deficiency
• Lightness not chromaticity dominates variation in natural scenes
• Small gamut and uneven frequencies of colored surfaces limit information available
• Red-green color deficient vision is little disadvantaged in natural environments

## Graphical abstract

**Figure:**
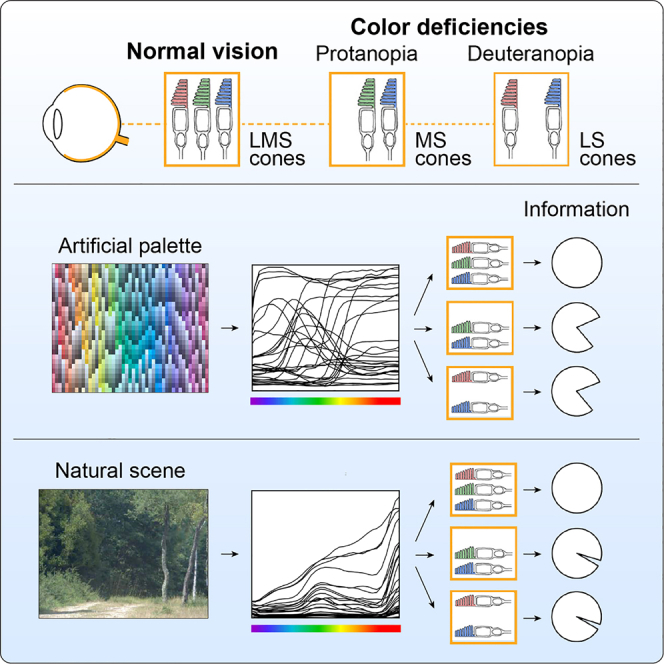

## Introduction

Not everyone has the same color vision. Data from European populations suggest about 8% of men have difficulty with red-green color discriminations and about 0.4% of women are similarly affected.^1^ These proportions reflect the genetic origins of most color vision deficiencies. Changes in the cone opsin gene on the X-chromosome array may result in the absence of the long-wavelength-sensitive (L) or medium-wavelength-sensitive (M) cone photoreceptor photopigments or variations in their spectral positions, which, depending on their expression, give rise to the deficiencies of dichromacy or anomalous trichromacy.^2^^,^^3^^,^^4^^,^^5^ In dichromacy, a single M pigment and an absent L pigment leads to protanopia and a single L pigment and an absent M pigment leads to deuteranopia.^6^ In anomalous trichromacy, two different M pigments and an absent L pigment leads to protanomaly and two different L pigments and an absent M pigment leads to deuteranomaly.^5^^,^^6^ Absence of the short-wavelength-sensitive (S) pigment, leading to tritanopia, is much rarer and has a different genetic origin.^4^^,^^5^ The various types of deficiency may be characterized behaviorally and clinically.^5^^,^^7^^,^^8^ Attempts to compensate for their effects with aids such as color filters have had limited success.^9^^,^^10^^,^^11^

Curiously, given the prevalence of red-green color vision deficiency, its impact on vision in the real world has not been quantified in any comprehensive way. As Carvalho et al. noted,^12^ most research has concentrated on a specific task, namely food selection, where, in primitive foraging, normal trichromacy offers an advantage for detecting red, orange, or yellow fruits among foliage,^13^^,^^14^^,^^15^^,^^16^^,^^17^^,^^18^^,^^19^ or young leaves against a background of mature leaves,^20^^,^^21^ or flower parts against leafy backgrounds.^22^ Other tasks have been considered, for example, detecting blushing or blanching, where normal trichromacy aids social signaling^23^^,^^24^; object detection, where slowly changing targets are more difficult to detect in dichromacy^25^; and breaking camouflage, where dichromacy offers a compensatory advantage with some scenes and targets,^26^^,^^27^ but not with others,^28^^,^^29^ or has a complex role.^30^^,^^31^

In yet other tasks, red-green color vision deficiency has been found to have less effect than expected on the discrimination of objects with spectral reflectances drawn from natural scenes,^32^ once their frequencies of occurrence are accounted for.^11^^,^^33^ It also has little effect on the judgment of the constancy of surface colors under different lights (illuminant color constancy) with surface spectral reflectances drawn from the approximately perceptually uniform Munsell Book of Color^34^^,^^35^^,^^36^ or from natural scenes.^35^^,^^37^^,^^38^^,^^39^ It additionally has little or no effect on memory for colored scenes^40^ or on discriminating typical illumination changes.^41^

The difficulty with assessing these tasks and their differing outcomes is that there is no straightforward way to decide on their relative importance or on how they relate to vision more generally in natural environments, and any selective genetic pressure they may, or may not, exert.^4^^,^^12^

The aim of this study was to take a more comprehensive approach to quantifying the impact of color vision deficiency. It entails estimating computationally the information available about the colored surfaces in scenes viewed by observers modeled with and without color vision deficiency. Information is understood in the sense of Shannon, namely as a reduction of uncertainty,^42^^,^^43^ and is usually measured in binary digits or bits, where one bit corresponds to a gain or loss by a factor of two. As explained later, it can be related to the effective number of surfaces in a scene that can be distinguished from each other by virtue of their reflecting properties.^44^ Importantly, it depends on the composition of the whole scene rather than on a subset of particular spectra either from the scene or recruited separately.

This approach requires only the spectral properties of the light reflected from each point in a sample of points from a scene, not the local or larger spatial features they may define, for example, texture, shape, location, and proximity to other spatial features, all of which entail assumptions about postreceptor processing in normal and color deficient vision.^45^ Some of these issues have been addressed elsewhere, for example, in considering the organization of the cone mosaic,^46^^,^^47^^,^^48^ the nature of postreceptor coding,^49^^,^^50^^,^^51^ and how it might be modified in dichromacy.^52^

**Figure 1:**
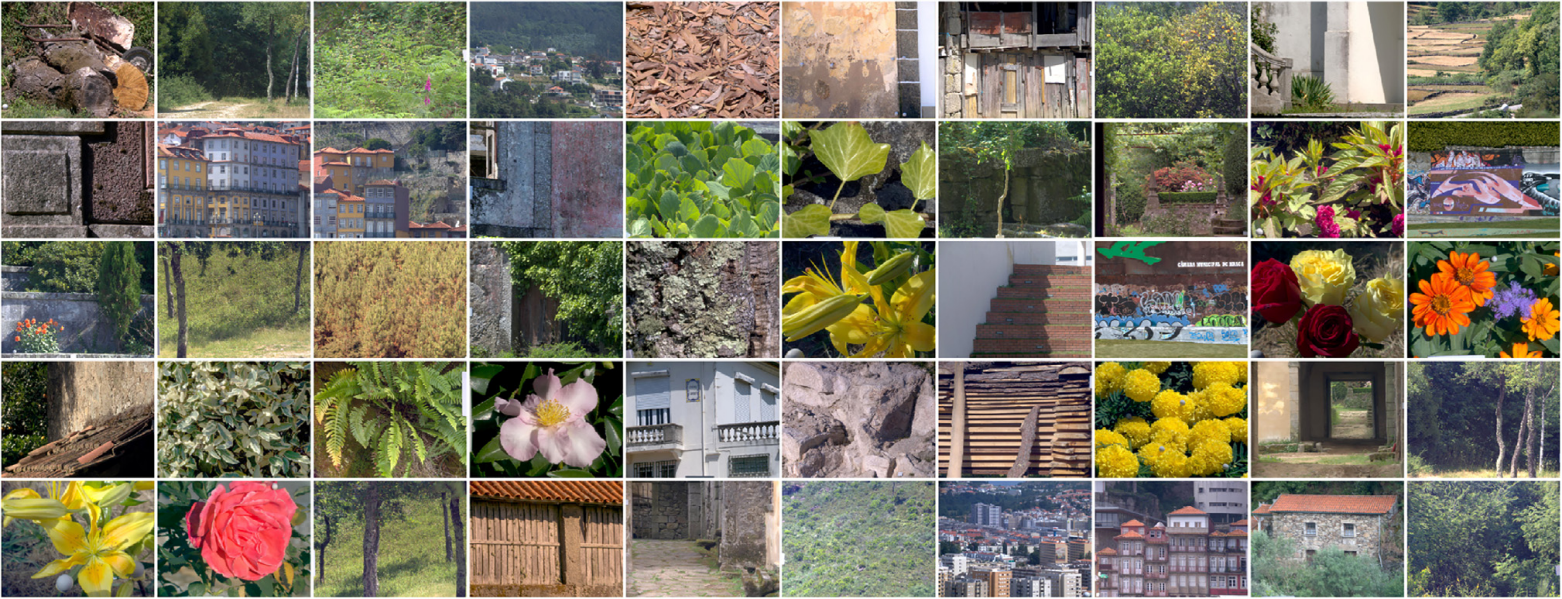
Color images of 50 natural scenes from one of the hyperspectral radiance image sets used here and in previously published works^53^^,^^57^ Each color image is rendered as an sRGB image^58^ with adjusted tonal range.

Scene data were taken from two independent sets of 50 and 100 hyperspectral radiance images of representative natural scenes.^53^^,^^54^ They were considered natural in the sense of being part of everyday outdoor environments, with a variety of undeveloped and developed land cover,^55^^,^^56^ by contrast with laboratory and virtual constructs. Figure 1 shows images of one of the sets.

To provide a reference level of performance, estimates of the information available with and without color vision deficiency were obtained from subsets of spectra that were more evenly distributed, that is, physically more uniform, than the spectra in individual scenes, and when pooled, covered much larger color gamuts. Since physical uniformity is not the same as perceptual uniformity, estimates were also obtained from the Munsell Book of Color and the Swedish Natural Color System,^59^ both of which are approximately perceptually uniform.

It was found that estimated information losses with natural scenes were much less than the information available, an outcome attributed to the large lightness variation within scenes, small chromaticity variation, limited color gamut, and uneven frequencies of different colored surfaces.

## Results

Information losses ΔI in red-green color vision deficiency are reported first, then comparisons with the colorimetric properties of natural scene images. The calculation of ΔI is described in STAR Methods.

### Size of information losses

**Figure 2:**
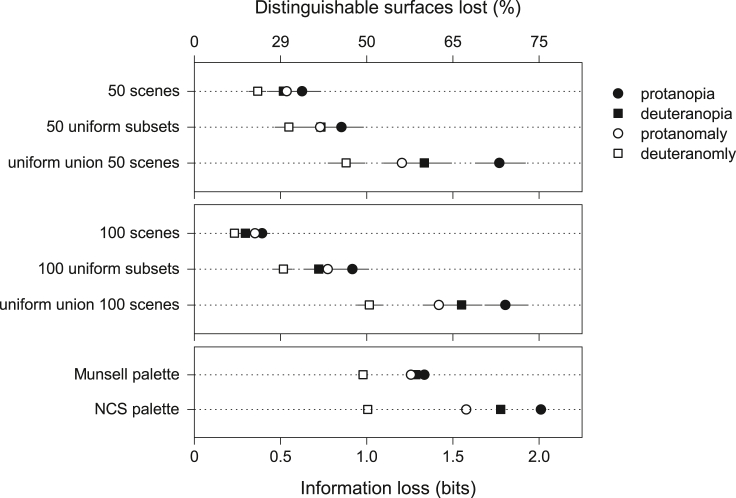
Losses of information in red-green color vision deficiency Symbols show the mean estimated losses ΔI in mutual information^43^ in protanopia, deuteranopia, protanomaly, and deuteranomaly. The top panel is for means over 50 natural scenes,^53^ then over 50 approximately uniform subsets of spectra from those scenes, and then for an approximately uniform union of spectra from all 50 scenes, maximizing the gamut available, as detailed in STAR Methods. The middle panel is the same but for the set of 100 natural scenes.^54^ The bottom panel is for the approximately uniform Munsell^60^ and NCS^59^ color palettes. Cone noise was assumed to be 2%. The bottom scale is linear in bits and the same in each panel; the upper scale is nonlinear and indicates the corresponding percentage reduction in the effective number of distinguishable surfaces. Horizontal bars mark 95% confidence intervals (CIs).

Figure 2 shows mean estimated information losses ΔI in protanopia, deuteranopia, protanomaly, and deuteranomaly. The top panel is for means over 50 natural scenes, then over 50 approximately uniform subsets of spectra from those scenes, and then for an approximately uniform union of spectra from all 50 scenes, maximizing the gamut available, as detailed in STAR Methods. The middle panel is the same but for the set of 100 natural scenes. The bottom panel is for the approximately uniform Munsell and NCS palettes. Information estimates assumed 2% cone noise. Other noise levels are dealt with later.

In the top panel, with the set of 50 scenes, mean information losses were 0.4–0.6 bits across the four types of color vision deficiency, equivalent to 6%–11% of the mean information available in normal trichromacy, about 5.9 bits. Losses in the effective number of distinguishable surfaces were proportionally greater, namely 25%–35%, because this number scales with the exponent of the information (STAR Methods). With the 50 approximately uniform subsets of spectra from the scenes, mean information losses increased from 0.4–0.6 bits to 0.5–0.9 bits, and with the approximately uniform union of spectra from all 50 scenes, to 0.9–1.8 bits.

In the middle panel, with the set of 100 scenes, the pattern was closely similar to that with the set of 50 scenes, but mean losses were less, namely 0.2–0.4 bits, equivalent to 4%–7% of the information available in normal trichromacy, about 5.4 bits. Losses in the effective number of distinguishable surfaces were 15%–25%. With the 100 approximately uniform subsets of spectra from the scenes, mean losses increased from 0.2–0.4 bits to 0.4–0.8 bits, and with the approximately uniform union of spectra from all the 100 scenes, to 0.9–1.7 bits.

In the bottom panel, with the Munsell and NCS palettes, the ranges of mean losses overlapped the ranges with the approximately uniform unions of spectra. The differences between the Munsell and NCS palettes seem likely to reflect their different chromatic structures.^61^^,^^62^^,^^63^

The presence of vegetation in scenes had a small effect. When the set of 50 scenes was divided into those that were either mainly vegetated or mainly non-vegetated,^64^ mean losses were about 0.1 bits larger with mainly vegetated scenes than with mainly non-vegetated scenes.

Corresponding estimates of mean information losses with smaller and larger levels of cone noise are available in Figures S1 and S2. The pattern of losses was largely preserved, that is, the smallest mean losses occurring with individual scenes and the greatest mean losses with approximately uniform unions of scenes and with color palettes. Common to all scenes, spectra, and palettes, information losses were least in deuteranomaly, greatest in protanopia, and intermediate in deuteranopia and protanomaly.

### Color variation

**Figure 3:**
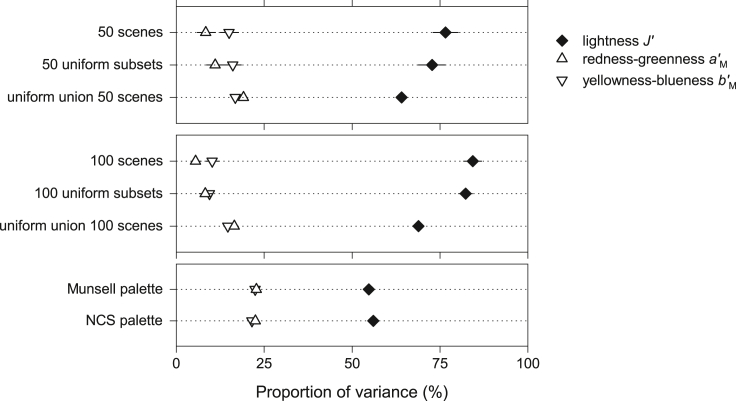
Color variance in scenes, subsets of spectra, and color palettes accounted for by colorimetric properties Symbols show the mean proportions of total color variance due to lightness $J^{\prime }$, redness-greenness $a_{M}^{'}$, and yellowness-blueness $b_{M}^{'}$ in the approximately uniform color space CAM02-UCS.^68^ The organization of the panels is the same as in Figure 2. Horizontal bars mark 95% confidence intervals.

Although measures of information and variation capture different properties of distributions,^65^^,^^66^ it is useful to quantify the variation in the conventional color characteristics of scenes^67^ to aid interpretation. Figure 3 shows mean color variance in scenes partitioned across the colorimetric correlates of lightness $J^{\prime }$, redness-greenness $a_{M}^{'}$, and yellowness-blueness $b_{M}^{'}$ in the approximately uniform color space CAM02-UCS.^68^ The organization of the panels is the same as in Figure 2.

Consistent with previous analyses of colored surfaces in natural scenes,^49^^,^^69^ lightness variation dominated. Averaged over the set of 50 scenes, it accounted for 77% (CI 73%–80%) of the total variance, with the remaining 23% (CI 20%–27%) of chromatic variance consisting of 15% due to yellowness-blueness and 8% due to redness-greenness (percentages have been rounded). With approximately uniform subsets of scene spectra, chromatic variance increased to 27% and with an approximately uniform union of spectra from all 50 scenes to 36%.

Results were similar with the set of 100 natural scenes but with a larger mean lightness variance of 84% (CI 82%–87%) and a smaller chromatic variance of 16% (CI 13%–18%), which presumably contributed to the smaller information losses in Figure 2. The presence of sky in many of the images seems not to be a factor, for when the upper halves of the scenes were removed, the maximum mean information losses were less than 0.03 bits. With approximately uniform subsets of scene spectra, chromatic variance increased to 18% and with an approximately uniform union of spectra from all 100 scenes to 31%.

With the approximately uniform Munsell and NCS palettes, chromatic variance was 45% and 44%, respectively.

Do these trends match those for losses of information in Figure 2? There is ordinal consistency in that with the larger chromatic variance in subsets of spectra and still larger with color palettes, the sizes of the mean information losses are correspondingly larger. On the other hand, the chromatic variance in scenes is small (and redness-greenness variance even smaller), which raises the general question of its relevance to information losses.

### Test of chromatic contribution

One way to assess the contribution of chromatic variation is to remove its effects. To this end, information losses in dichromacy were compared with those in monochromacy, where with just one cone class, no chromatic information was available.

**Figure 4:**
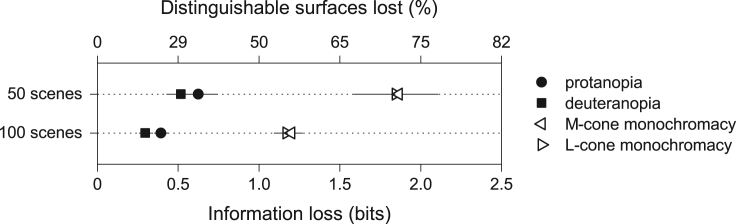
Losses of information in dichromacy and monochromacy Symbols show the mean estimated losses ΔI in mutual information^43^ in protanopia and deuteranopia taken from Figure 2 and in M- and L-cone monochromacy. Other details as for Figure 2.

Figure 4 shows mean estimated information losses ΔI for protanopia and deuteranopia, taken from Figure 2, and for M-cone monochromacy and L-cone monochromacy, each with 2% cone noise. Results are shown for the set of 50 scenes and the set of 100 scenes.

The losses with a single cone class were 3.0–3.9 times more than with two cone classes, confirming the importance of chromatic information in individual scenes. This result, though, is not inconsistent with the relatively small chromatic variance in Figure 3, since the mean losses of 0.2–0.6 bits shown in Figure 2 remained much smaller than the mean available information of 5.4–5.9 bits in normal trichromacy, mentioned earlier.

### Prediction by redness-greenness variance

A more specific parametric test of the contribution of chromatic variation is whether information losses increase as the redness-greenness variance in individual scenes increases. The outcome is not foregone since there is no consistent relationship between information and variance^65^^,^^66^ and both are affected by differences in frequency distributions,^43^^,^^70^ in this case of the colored surfaces in scenes.

**Figure 5:**
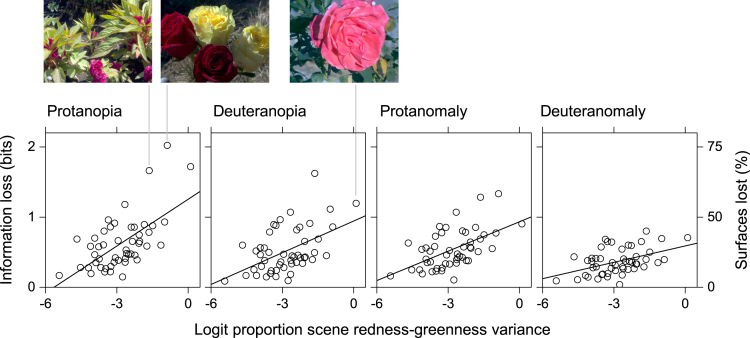
Losses of information in red-green color vision deficiency as a function of redness-greenness variation in 50 natural scenes Symbols show the estimated losses ΔI in mutual information in protanopia, deuteranopia, protanomaly, and deuteranomaly plotted against the logit of the proportions of total variance due to redness-greenness $\mathrm{var}\left(a_{M}^{'}\right)$ in the approximately uniform color space CAM02-UCS.^68^ Cone noise was assumed to be 2%. The left vertical scale is linear in bits and the same in each panel; the rightmost vertical scale is nonlinear and indicates the corresponding percentage reduction in the effective number of distinguishable surfaces. The straight lines are linear regressions. The thumbnail images show three scenes with the largest estimated information losses.

Figure 5 shows for the set of 50 scenes the estimated information losses ΔI with 2% cone noise plotted against the proportions of total variance in each scene due to redness-greenness $a_{M}^{'}$, linearized with the logistic (logit) transformation.^71^ The straight lines are linear regressions. The gradients are all positive but as with the mean losses in Figure 2, they decline progressively, that is, for protanopia 0.22 (CI 0.11–0.31), deuteranopia 0.15 (CI 0.07–0.22), protanomaly 0.14 (CI 0.08–0.20), and deuteranomaly 0.08 (CI 0.04–0.12). None of the confidence intervals contained zero. Similarly with the set of 100 scenes (Figure S3).

Notwithstanding the reliability of the trends, the proportions of variance *R*^2^ accounted for by the regressions are modest. From protanopia to deuteranomaly, values varied from 38% to 21%, with the set of 50 scenes, and from 43% to 38% with the set of 100 scenes. The regressions on lightness $J^{\prime }$ and on yellowness-blueness $b_{M}^{'}$ (not shown here) were smaller still, not more than 13% and 4%, respectively, for both sets of scenes.

### Prediction by chromatic axis

A different test of the effects of chromatic variation is whether information losses vary predictably with the direction of most chromatic variation in each scene, and in particular, whether the losses are maximum when the direction coincides with the confusion axis in protanopia and in deuteranopia.^72^^,^^73^ As before, the distinction between information and variation^65^^,^^66^ should be kept in mind.

**Figure 6:**
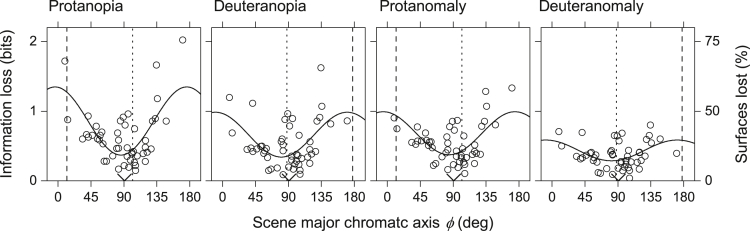
Losses of information in red-green color vision deficiency as a function of the direction of the major axis of chromatic variation in a set of 50 natural scenes Symbols show the estimated losses ΔI in mutual information in protanopia, deuteranopia, protanomaly, and deuteranomaly plotted against the directions *φ* of the major chromatic axes (STAR Methods) in the approximately uniform color space CAM02-UCS,^68^ with ϕ = 0° (identical with ϕ = 180°) corresponding to the redness-greenness axis $a_{M}^{'}$. Cone noise was assumed to be 2%. The left vertical scale is linear in bits and the same in each panel; the rightmost vertical scale is nonlinear and indicates the corresponding percentage reduction in the effective number of distinguishable surfaces. The continuous curves are linear circular regressions.^74^ The vertical dashed lines indicate the directions of the dichromatic confusion axes and the dotted lines the orthogonal directions.^76^ The circular mean directions^74^ of the major chromatic axes are marked by a “v” on the horizontal axis.

Figure 6 shows for the set of 50 scenes the estimated information losses ΔI with 2% cone noise plotted against the directions ϕ of the major chromatic axis of variation in each scene. The solid curves are linear circular regressions,^74^^,^^75^ and the vertical dashed lines indicate the directions of the estimated dichromatic confusion axes and the dotted lines the directions orthogonal to those axes (STAR Methods).

The maxima and minima of the circular regressions fall close to the expected directions, though for both protanopia and protanomaly, there are reliable differences of 16° (CI 8°–22°) and 18° (CI 7°–25°), respectively. From protanopia to deuteranomaly, values of *R*^2^ varied from 46% in protanopia to 19% in deuteranomaly.

It is possible that the estimated directions of maximum information loss were biased by the clustering of the major chromatic axes around 90°, the yellowness-blueness axis $b_{M}^{'}$. A control is provided by results with the set of 100 scenes where the major chromatic axes were clustered more around 70° (Figure S4). The estimated directions of maximum loss were little affected.

## Discussion

A focus on specific visual tasks can lead to conflicting interpretations of the impact of color vision deficiency in the real world, ranging from being disadvantageous to broadly neutral or even advantageous. All tasks, however, are contingent on the information available. The present analysis suggests that this information is only a little less in red-green color vision deficiency than in normal trichromacy, by between 4% and 11%, depending on cone noise and the type of color vision deficiency. There are multiple contributory factors, some of which are explored in the following.

### Degree of color vision deficiency

Across the four types of red-green color vision deficiency, estimated information losses with the 50 natural scenes ranged from about 0.4 to 0.6 bits out of about 5.9 bits available in normal trichromacy. Estimated losses with the 100 natural scenes were still smaller, from about 0.2 to 0.4 bits out of about 5.4 bits available. The differences between the two sets of scenes seem not to be due to the presence of sky in the set of 100 scenes but to their smaller chromatic variation.

The types of color vision deficiency had predictable effects. Losses were least in deuteranomaly with the largest gap between normal and hybrid L pigment peaks at 559 nm and 549 nm, and they were greatest in protanopia with a normal M pigment peak at 530 nm.^5^ Losses were intermediate and similar in deuteranopia, with a normal L pigment peak at 559 nm, and in protanomaly with normal and hybrid M pigment peaks at 530 and 536 nm. The S pigment, with peak at 426 nm, was assumed to be unaltered. These values were chosen to represent the larger discrete differences in pigment peaks,^5^^,^^77^ which also depend on the frequency, type, and expression of opsin gene arrangements^78^ as well as on variations in the density of the lens pigment and macular pigment at the fovea, and in the optical density of the cone photopigment.^79^^,^^80^ The finding that protanopic losses were always greater than deuteranopic losses confirms the advantage afforded by the approximately 30-nm shift in peak sensitivity from the normal M pigment to the normal L pigment.^81^

### Role of color gamut and frequency

Early conjectures about the small range of chromaticities in outdoor scenes were based on data from visual spot matching^82^ and photographic colorimetry,^83^ later confirmed by more comprehensive hyperspectral scene imaging.^69^^,^^84^^,^^85^^,^^86^ Yet as Figure 2 showed, it is not just the size of the gamuts of these scenes but the frequencies of occurrence of colors within those gamuts that govern the information available. Recall that as the frequencies of spectra were made more uniform within scenes, the estimated information available increased and so did the information losses in color vision deficiency, by 1.4–2.0 times. When gamuts were maximized by taking the union of the spectra in scenes and then made more uniform still, the information available increased further and so did the information losses, by 2.4–4.2 times (Figure 2). With the approximately uniform color palettes of the Munsell and NCS sets, estimated information losses were also larger than with individual scenes, by 2.1–5.1 times. Whether defined physically or perceptually, approximately uniform sets of spectra maximizing the gamut available appear to provide an upper bound on information losses.

### Chromatic variation vs. lightness variation

One reason for the smaller chromatic variation within scenes and the larger lightness variation, amounting to 80% on average, may be the way natural light is distributed within the environment.^49^^,^^87^^,^^88^^,^^89^^,^^90^ Nearby objects and overhead foliage^91^^,^^92^ can each interrupt the direct beam to produce cast shadows where the reduction in radiant intensity exceeds the change in spectral composition. Another reason may be the nature of local surface geometry.^87^ Constant surface orientation usually persists over shorter distances than material identity so that changes in reflected intensity exceed those in spectral composition, given that natural surfaces tend to be neither Lambertian nor specular^87^ (a potential counter to this explanation is the observation that natural scenes may contain large-amplitude chrominance variations at high spatial frequencies^85^).

As indicated earlier, though, this does not mean that information losses in color vision deficiency are small simply because there is little chromatic information to lose. When the chromatic component was effectively removed, leaving only lightness information, estimated losses increased by three to four times. The limitations of lightness information were previously noted in an experiment^33^ where dichromatic observers discriminated real three-dimensional objects whose spectral reflectances had the same frequencies of occurrence as in some of the scenes in this analysis.

The variation in redness-greenness in scenes also had its predictable effects on estimated information losses, which increased both as the proportion of the variance in scenes increased and as the direction of the major chromatic axis approached the dichromatic confusion axes. Nevertheless, as a predictor, chromatic variation in either magnitude or direction accounted for less than half of the estimated information losses.

### Outliers

Although information losses may be small when averaged over natural scenes, it is of course possible to find individual scenes where losses are much greater. Three instances from the set of 50 scenes are shown by the thumbnail images in Figure 5. Two of these scenes had the greatest proportions of redness-greenness variance and were among the more colorful images. Likewise for the set of 100 scenes (Figure S3). Such scenes may not only be disadvantageous for color deficient vision but lead to failures of color constancy in normal trichromacy.^57^^,^^93^

### Other information estimates

There have been few other studies of the information available from natural scenes at the level of cones. The most relevant is an analysis by Lewis and Zhaoping^81^ in which information was used to derive optimum spectral locations of L, M, and S cone pigments. Direct comparison with the present findings is difficult since scene spectra were represented^81^ by principal components with weights drawn from truncated Gaussian distributions (spectra from individual objects and fruits were similarly represented). That said, data from their Figures 6 and 7 suggest somewhat smaller losses in dichromatic vision, namely about 0.2 or 0.3 bits in deuteranopia and 0.1 or 0.3 bits in protanopia, depending on the noise regime. Estimates with approximately uniform frequency distributions were not available.

Higher-level informational issues have also been examined, for example, the estimated information preserved from scenes under illuminant changes^94^^,^^95^; the coding efficiency of different color spaces^96^; the effects of fluctuating environmental illumination^97^; and the information extracted at different levels of the visual pathway.^98^

### One or many tasks?

If, as argued here, red-green color deficient vision is little disadvantaged in natural environments, it may be construed as evidence of a larger relaxation of the pressure to maintain trichromacy in modern human societies.^28^^,^^99^ But this is not to say that scene information does not bear on specific tasks requiring color vision, implicitly, or explicitly.^100^ Consider the exemplary task of selecting fruit from foliage in the wild. Making that selection involves multiple activities.^18^ Together with initially searching a complex optical environment,^87^^,^^101^ an individual navigating toward an area of interest under direct and diffuse illumination^89^^,^^97^^,^^102^ needs to continuously accommodate changes in the intensity and spectrum of light from reflecting surfaces^90^ as relative orientations change^87^ and specularities appear and disappear, all of which affect the information available, and eventual success in the task.

### Limitations of the study

There are two general limitations to this study, in addition to those already mentioned. First, the magnitudes of the estimated information losses in color deficient vision of 4%–11% depend on the practical minimum level of 2% noise assumed in cones.^103^ For levels of noise below and above 2%, losses were, respectively, larger and smaller (Figures S1 and S2). The advantage of natural scenes was, however, retained.

Second, the information estimates remain theoretical and concern solely the information from colored surfaces in scenes. No account was taken of how observers might use that information to guide fixations or interrogate scenes. Encouragingly, the estimate for dichromats of effectively about one-third fewer distinguishable surfaces (average data from Figure 2, top panel) is similar to a reduction in dichromatic observers’ experimental discrimination of object spectral reflectances with natural frequencies of occurrence.^33^

### Conclusion

Despite difficulties with some discrimination tasks, individuals with red-green color vision deficiency lose little information from natural environments. But there is no single determining factor. Rather, it is the large variation of lightness within scenes, the small variation in chromaticity, especially in redness-greenness, the limited color gamut, and the uneven frequencies of different colored surfaces that all lessen the impact of color vision deficiency.

## STAR★Methods

### Key resources table

**Table:** 

REAGENT or RESOURCE	SOURCE	IDENTIFIER
**Software and algorithms**		

50 hyperspectral radiance images	Foster et al. (2022)^57^	https://doi.org/10.48420/14877285
100 hyperspectral radiance images	Arad and Ben-Shahar (2016)^54^	https://icvl.cs.bgu.ac.il/hyperspectral/
Spectral reflectances of the matt Munsell set	Parkkinen et al. (1989)^104^	https://sites.uef.fi/spectral/munsell-colors-matt-spectrofotometer-measured/
Human cone pigment spectral sensitivities	Stockman and Sharpe (2000)^105^	http://cvrl.ioo.ucl.ac.uk/
Cone pigment shift routine	Foster, D.H. (2010)^106^	https://doi.org/10.5281/zenodo.8121909
Offset version of the Kozachenko-Leonenko estimator of mutual information	Marín-Franch et al. (2022)^107^	https://github.com/imarinfr/klo

### Resource availability

#### Lead contact

Requests for resources or information should be directed to the lead contact, David H. Foster (d.h.foster@manchester.ac.uk)

#### Materials availability

This study did not generate new materials.

### Experimental model and study participant details

#### Model

Simulations were based on a model of human cone photoreceptor activity in which light reflected from each point of a scene gives rise to excitations of L, M, and S cones, with normal, hybrid, or absent photopigments, which in turn provide information about the contents of the scene. In brief, let L(u,v;λ) be the reflected radiance, indexed by spatial coordinates *u*, *v* and wavelength *λ*, and let $S_{L}\left(\lambda \right),S_{M}\left(\lambda \right),S_{S}\left(\lambda \right)$ be, respectively, the long-, medium-, and short-wavelength-sensitive cone spectral sensitivities, measured at the cornea, that is, incorporating preceptor absorption.^105^^,^^108^ Then at each point (u,v) in the scene, the corresponding cone excitations $q_{L},q_{M},q_{S}$ are given by

$$\begin{array}{l} q_{L}=\int L\left(\lambda \right)\;S_{L}\left(\lambda \right)\;d\lambda \text{,}\\ q_{M}=\int L\left(\lambda \right)\;S_{M}\left(\lambda \right)\;d\lambda \text{,}\\ q_{S}=\int L\left(\lambda \right)\;S_{S}\left(\lambda \right)\;d\lambda \text{,} \end{array}$$

where integration is over the visible range.^109^ How scene information was calculated from these cone excitations with normal, hybrid, or absent photopigments is described later. Simulations were implemented in the MATLAB computing environment (Version R2022a, The MathWorks, Inc., Natick, MA).

### Method details

Some of the detail in this and following sections is reproduced from previous work.^10^^,^^57^^,^^97^

#### Scene spectral data

One set of scene images consisted of 50 hyperspectral radiance images of outdoor scenes drawn from the main land-cover classes. Of the 50 scenes, 30 were classified^55^^,^^56^ as predominantly vegetated, containing woodland, shrubland, herbaceous vegetation (e.g., grasses, ferns, flowers), and cultivated land (fields), and 20 were classified as predominantly nonvegetated, containing barren land (e.g., rock or stone), urban development (residential and commercial buildings), as well as farm outbuildings and painted or treated surfaces. Details of image acquisition, calibration, and processing as radiance images are described elsewhere.^64^^,^^109^ Each radiance image had dimensions ∼1344 × 1024 pixels and a spectral range 400–720 nm sampled at 10-nm intervals, giving 33 values at each pixel. The angular subtense of each scene at the hyperspectral camera was approximately 6.9° × 5.3°. To reduce pixel-pixel correlations and non-imaging noise,^57^ images were downsampled by spatial averaging over 2 × 2 pixels. Figure 1 shows sRGB^58^ rendered color images of the scenes. Almost all the scenes contained color, quantified by the ratio of chroma to lightness.^61^ The median ratio was 0.25 and only three scenes had ratios less than or equal to 0.05. None of the images contained sky.

The other set of images consisted of 100 hyperspectral radiance images of outdoor scenes, classified as urban (residential and commercial), suburban, rural, and plant life. Details of image acquisition are described elsewhere.^54^ Each image had dimensions ∼1392 × 1300 pixels and a spectral range 400–1000 nm sampled initially at ∼1.25 nm intervals and then downsampled to 400–700 nm at 10 nm intervals, giving 31 values at each pixel. For this analysis, a correction was made for the estimated level of dark noise in the images, after which they were downsampled by spatial averaging over 2 × 2 pixels. All the scenes contained color, and the median ratio of chroma to lightness was 0.25 with no scenes having ratios less than 0.10. About 80 of the 100 images contained sky.

For comparison, sets of physically more uniformly distributed spectra in the (bounded) space of radiance spectra were generated in two ways. First, approximately uniform subsets of spectra were obtained from each of the 50 images in the following way (notation adapted from an earlier account^109^). Let $l_{ij}\left(\lambda \right)$ denote the spectral radiance at wavelength λ and spatial position ${\left(u,v\right)}_{i}$, indexed by *i* in image *j* and let ${\left\{l_{i}\right\}}_{j}$ denote the set of all such spectra from image *j*, where *i* depends on *j*. Assume that the wavelength range is the same for each *i*, *j*. The required subset ${\left\{l_{i\left(k\right)}\right\}}_{j}$ of ${\left\{l_{i}\right\}}_{j}$, indexed by *k*, was then obtained by thinning,^110^ that is, by removing spectra $l_{r}$ from ${\left\{l_{i}\right\}}_{j}$ within a distance *d* of other spectra $l_{s}$ as *d* was progressively increased up to a limit for which stable information estimates could be obtained.^111^ The distance *d* between two spectra was defined by the sup metric, $d\left(l_{r},l_{s}\right)=\underset{\lambda }{\text{sup}}\left(\left|l_{r}\left(\lambda \right)-l_{s}\left(\lambda \right)\right|\right)$. The procedure was repeated for each image *j.* Second, a single, approximately uniform subset of spectra maximizing the gamut available was obtained from the thinned sets ${\left\{l_{i\left(k\right)}\right\}}_{j}$ by taking their union over all *j* to form the set $\left\{l_{i\left(k\right),j}\right\}$, and then applying thinning once more. A similar procedure was followed with the set of 100 images.

#### Approximately uniform color palettes

Two large gamut, approximately perceptually uniform sets of reflectance spectra were taken from the set of matt Munsell chips^60^ and the set of Natural Color System (NCS) samples.^59^ Both palettes span a wide range of chromaticities and lightnesses,^61^^,^^62^ and subsets of the Munsell palette have been used in practical tests of color discrimination ability,^8^ most notably in the Farnsworth-Munsell 100-Hue Test.^112^ Despite the artificial construction of the Munsell set, its dimensionality is similar to that of natural reflectance spectra.^113^^,^^114^^,^^115^

Even for a normal trichromatic observer, however, the approximate uniformity of each of the two color palettes holds only imperfectly and mainly locally.^116^^,^^117^^,^^118^ The designs of the two systems are fundamentally different,^61^^,^^62^ and systematic perceptual differences have been reported between the two,^63^ and between their gamuts and the gamuts of natural reflectance spectra.^119^

For this analysis, the spectral reflectances of the Munsell set were obtained from measurements by Parkkinen et al.,^104^ and the spectral reflectances of the NCS palette were recorded in-house with a Konica Minolta CM-2600d spectrophotometer.^119^ Radiant spectra from the surfaces were generated by taking the product of each reflectance spectrum and a 6500 K daylight illuminant spectrum.^68^ The set of Munsell radiance spectra was treated as a single scene, as was the set of NCS radiance spectra.

#### Scene color attributes

To characterize the color variation in scenes, spectra, and palettes, each was mapped into the approximately uniform color space CAM02-UCS,^68^ which provides colorimetric correlates of lightness $J^{\prime }$, redness-greenness $a_{M}^{'}$, and yellowness-blueness, $b_{M}^{'}$, analogous to the traditional *L*∗, *a*∗, *b*∗ of the less uniform color space CIELAB.^68^ Summary measures of the resulting distributions of points in CAM02-UCS included the mean ratio of chroma to lightness, i.e., ${\left[{\left(a_{M}^{'}\right)}^{2}+{\left(b_{M}^{'}\right)}^{2}\right]}^{1/2}/J^{\prime }$; the proportions of total variance accounted for by $J^{\prime }$, $a_{M}^{'}$, and $b_{M}^{'}$, e.g., for lightness, $\mathrm{var}\left(J^{\prime }\right)/\left[\mathrm{var}\left(J^{\prime }\right)+\mathrm{var}\left(a_{M}^{'}\right)+\mathrm{var}\left(b_{M}^{'}\right)\right]$; and the major chromatic axis of the distribution, that is, the direction of most variance in the chromatic plane determined by a principal component analysis of $\left(a_{M}^{'},b_{M}^{'}\right)$ values. In regressions on the proportions of variance, the latter were linearized with the logistic (logit) transformation.^71^

For clarity, these colorimetric quantities and the color space CAM02-UCS were used solely for the conventional descriptions of scenes, subsets of spectra, and color palettes and were not part of the calculations of the information estimates for normal trichromatic or color deficient vision.

#### Confusion loci

Confusion loci describe the sets of colors in the chromatic plane that a dichromatic observer can match by luminance adjustments only.^72^^,^^120^ In the CAM02-UCS $\left(a_{M}^{'},b_{M}^{'}\right)$ plane, these loci generally form curves, and their axial directions^74^ at the origin were estimated numerically by mapping the tangent vector at the origin in the CIE 1931 (*x*, *y*)-chromaticity diagram.^72^^,^^120^ The angles, measured anticlockwise from the redness-greenness axis $a_{M}^{'}$, corresponding to 0°, were approximately 12°, 178°, and 114° for protanopia, deuteranopia, and tritanopia, respectively. The directions orthogonal to these confusion axes, ideally optimal for discrimination,^76^ were approximately 102°, 88°, and 24°, respectively.

#### Cone spectral sensitivities

Radiance spectra were converted^109^ into L, M, and S cone excitations based on the Stockman and Sharpe 2° cone spectral sensitivities, lens, and macular pigment data.^108^^,^^105^ Spectrally shifted absorption spectra were derived from a quadratic loess fit^121^ to the normal L, M, and S absorption spectra on a scale of radiance versus log wavelength, with optimal loess bandwidth determined by cross-validation.^106^ The normal L, M, and S pigments were assumed to have maximum sensitivities at approximately 559, 530, and 426 nm, respectively.^5^^,^^105^^,^^122^ In protanomaly, the normal and hybrid medium-to-long-wavelength cone pigments were assumed to have maximum sensitivities at approximately 530 and 536 nm, respectively, and in deuteranomaly, at 559 and 549 nm.^5^ The sizes of the hybrid shifts were intended to illustrate their range, not necessarily their prevalence. Optical density was taken to be constant.^79^^,^^123^

#### Cone noise

Cone signal processing was assumed to be limited by phototransduction noise,^47^^,^^124^ which varies linearly with background level over a wide range,^125^ though the extent of the relevant background may be difficult to specify.^126^ The noise distribution was modeled as a Gaussian process whose standard deviation (SD) at each point was specified relative to the cone excitation locally at that point. Previous simulations^10^ have found that this process produced similar informational dependencies as a Gaussian process with SD defined relative to the global mean and a Poisson process with an adjusted global mean. The choice of coefficient of proportionality, the Weber fraction, was guided by Stiles’ psychophysical increment threshold measurements,^103^ which yielded Weber fractions of 0.018, 0.019, and 0.087 for L, M, and S cones, respectively. For brevity, representative values of the relative SD are reported just for L cones, with values for M and S cones scaled appropriately.^10^ Results are presented for a relative SD of 0.02, taken as a practical minimum, but other values of 0.01 and 0.05 were also tested.

### Quantification and statistical analysis

#### Mutual information estimates

The information available from scenes, subsets of spectra, and color palettes was estimated with sample points drawn randomly and uniformly from the source data.^109^ The spectral radiances and the corresponding L, M, and S cone excitations, together with cone noise, were therefore treated as continuous random variables,^44^ respectively **X** and **Y** say, where **X** is 33-dimensional or 31-dimensional, depending on the hyperspectral image set, and **Y** is 3-dimensional or 2-dimensional, depending on the color vision deficiency. The amount of information that **Y** conveys about **X** is given by the mutual information, written I(X;Y), or I for short, and is defined in terms of Shannon differential entropies^43^ (other definitions exist^42^).

Mutual information was estimated numerically with an offset version^111^^,^^107^ of the Kozachenko-Leonenko *k*th-nearest-neighbor estimator,^127^^,^^128^ which converges relatively rapidly and accurately with increasing sample size.^111^^,^^107^ The number of sample points taken from each image in the set of 50 and set of 100 images was 10^4^ and the maximum available were taken from the color palettes, namely ∼10^3^. Resampling was used to test the stability of the estimates of I(X;Y). As in previous analyses,^109^ these estimates refer to the underlying continuous spectral radiance distributions, not the discrete hyperspectral images that represent them.

Mutual information is related by the inverse logarithm to the approximate number *N* of distinguishable surfaces or parts of surfaces in a scene, subset of spectra, or color palette, taking into account their different frequencies of occurrence.^44^^,^^86^ With I(X;Y) measured in bits, the inverse logarithm is to the base 2, so that *N* = 2^*I*(**X**;^ ^**Y**)^, sometimes referred to as the effective alphabet size,^43^^,^^129^ which, in the present context, is the effective number of distinguishable surfaces. Percentage reductions in *N* are expressed with respect to the number for a normal trichromatic observer.

Notice that I(X;Y) represents the information available at the level of cone receptors, not necessarily at successive postreceptor stages.^51^ Nonetheless, in the light of the data-processing inequality,^43^ the information that Y contains about X must either remain the same or decrease postreceptorally, since no postreceptor manipulation can increase it.

For each scene, subset of spectra, or palette, the loss of information was measured by the difference ΔI between the mutual information I available in normal trichromacy and the mutual information $I^{\prime }$ in color vision deficiency, that is, $\Delta I=I-I^{\prime }$.

#### Statistical analysis

Comparisons of means of data and of differences between means were based on 95% confidence intervals (CIs). Intervals were estimated with Efron’s percentile bootstrap^130^ method, which was used in nonparametric mode, thereby avoiding parametric assumptions about the form of the underlying population. It was implemented with the MATLAB function bootci, with 1000 bootstrap replications. Means and differences between means with estimated CIs are shown in Figures 2, 3, and 4 and in Figures S1 and S2, all using the same bootstrap procedure.

Linear regressions were implemented with the MATLAB function regress. Comparisons of estimated gradients were based on 95% CIs. Values are reported in the results section dealing with prediction by redness-greenness variance.

Linear circular regressions^74^^,^^75^ of data y on circular variables x, expressed in degrees, were implemented with the MATLAB model mdl = fitlm([cos(x∗pi/180), sin(x∗pi/180)], y). Axial data defined over 180°, such as chromatic axes, were converted to vectorial data defined over 360° by doubling and then reducing modulo 360°. After analysis, they were then transformed back to axial data.^74^^,^^75^ Comparisons of estimated directions were based on 95% CIs. Values are reported in the results section dealing with prediction by chromatic axis.

## Data Availability

• Data generated by this study are available from the lead contact upon request. • Original code has been deposited at Zenodo and is publicly available as of the date of publication. Other data and code listed in the key resources table are publicly available. • Any additional information required to reanalyze the data reported in this paper is available from the lead contact upon request.
