# Supplementary material for: Little information loss with red-green color deficient vision in natural environments
Source: iScience. 2023 Jul 18;26(8):107421. doi: 10.1016/j.isci.2023.107421 (PMC10428128; doi:10.1016/j.isci.2023.107421)
Supplement: Document S1. Figures S1–S4 [file mmc1.pdf]

iScience, Volume *26*

Supplemental information

Little information loss with red-green color deficient vision in natural environments

David H. Foster and Sérgio M.C. Nascimento

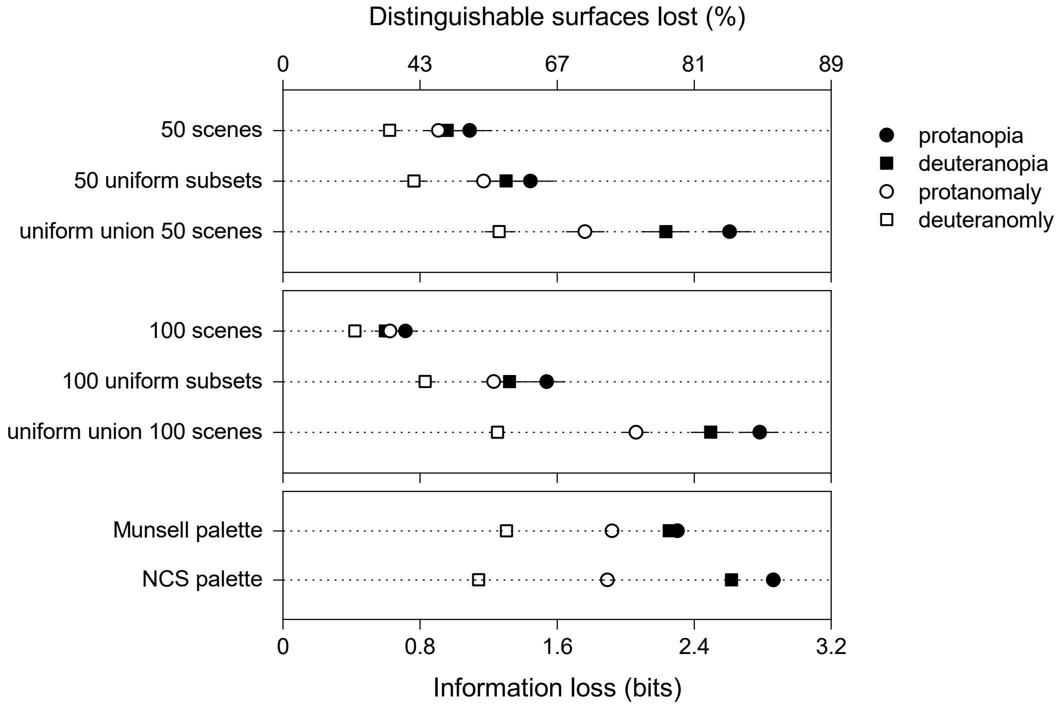

Figure S1. As for Figure 2 but with 1% cone noise. The common horizontal axis scale is adjusted for the data range.

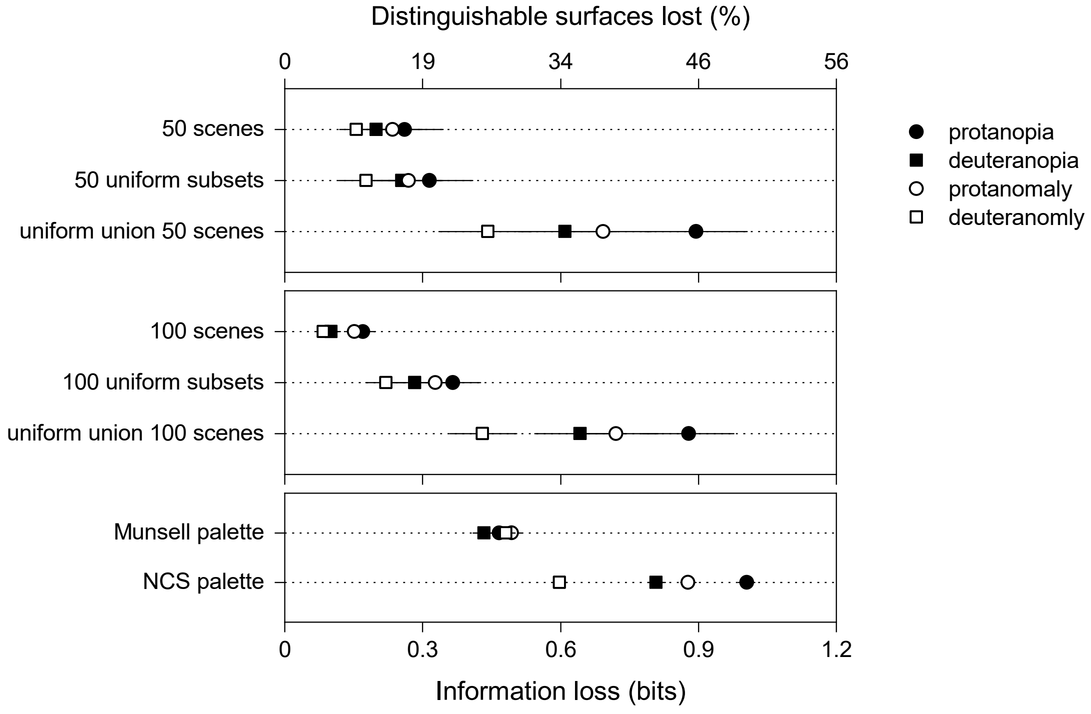

Figure S2. As for Figure 2 but with 5% cone noise. The common horizontal axis scale is adjusted for the data range

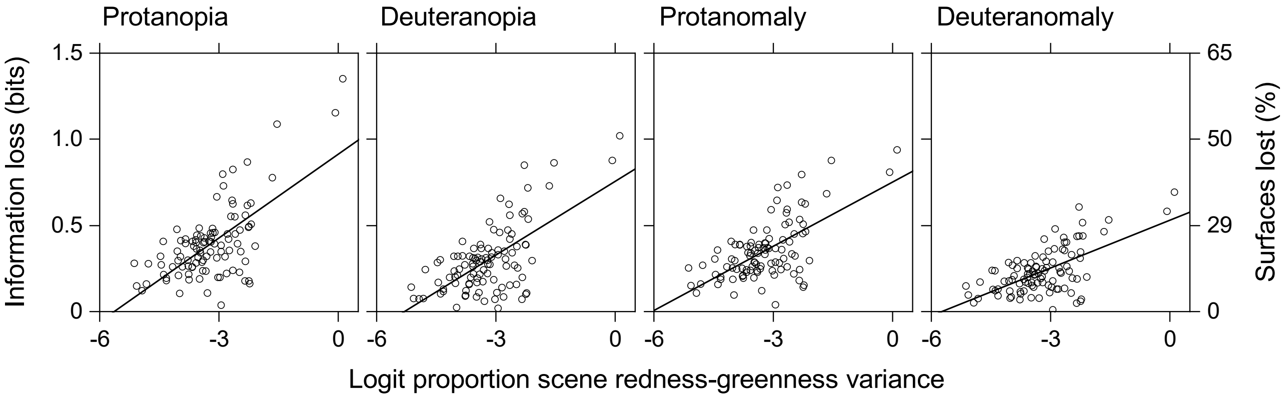

Figure S3. As for Figure 5 but for the set of 100 natural scenes [55]. The common vertical axis scale is adjusted for the data range.

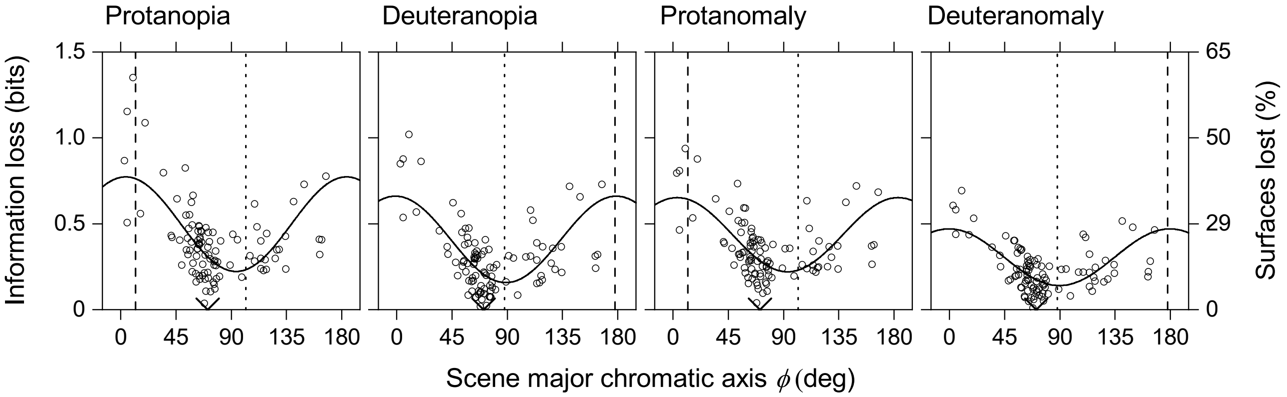

Figure S4. As for Figure 6 but for the set of 100 natural scenes [55]. The common vertical axis scale is adjusted for the data range.
